# Immunomodulatory and anticancer potential of Gan cao *(Glycyrrhiza uralensis* Fisch.) polysaccharides by CT-26 colon carcinoma cell growth inhibition and cytokine IL-7 upregulation in vitro

**DOI:** 10.1186/s12906-016-1171-4

**Published:** 2016-07-11

**Authors:** Peter Amwoga Ayeka, Yuhong Bian, Peter Githaiga Mwitari, Xiaoqian Chu, Yanjun Zhang, Rosette Uzayisenga, Elick Onyango Otachi

**Affiliations:** Tianjin University of Traditional Chinese Medicine, 312 Anshan Western Road, Nankai District, Tianjin 300193 People’s Republic of China; Department of Biological Sciences, Faculty of Science, Egerton University, PO BOX 536-20115, Egerton, Kenya; Center for Traditional Medicine and Drug Research, Kenya Medical Research Institute, P.O. Box 54840-00200, Nairobi, Kenya; School of Pharmacy, Mount Kenya University/Kigali campus, P.O BOX 5826, Kigali, Rwanda; Tianjin University of Traditional Chinese Medicine, 312 Anshan Western Road, Nankai District, Tianjin, 300193 People’s Republic of China

**Keywords:** *Glycyrrhiza uralensis*, Immunomodulatory, TCM, Anti-cancer, IL-7, Polysaccharides

## Abstract

**Background:**

Chinese licorice, (*Glycyrrhiza uralensis* Fisch.) is one of the commonly prescribed herbs in Traditional Chinese Medicine (TCM). Gancao, as commonly known in China, is associated with immune-modulating and anti-tumor potential though the mechanism of action is not well known. In this study, we investigated the in vitro immunomodulatory and antitumor potential of *Glycyrrhiza uralensis* polysaccharides fractions of high molecular weight (fraction A), low molecular weight (fraction B) and crude extract (fraction C).

**Methods:**

Cell proliferation and cytotoxicity was investigated using Cell Counting kit 8 (CCK-8) on Intestinal epithelial cell line (IEC-6) and Colon carcinoma cell line (CT-26). IL-7 gene expression relative to GAPDH was analysed using Real time PCR. The stimulation and viability of T lymphocytes was determined by Trypan blue exclusion assay.

**Results:**

*G.uralensis* polysaccharides did not inhibit proliferation of IEC-6 cells even at high concentration. The ED_50_ was found to be 100 μg/ml. On the other hand, the polysaccharides inhibited the proliferation of cancer cells (CT-26) at a concentration of ≤50 μg/ml. Within 72 h of treatment with the polysaccharides, expression of IL-7 gene was up-regulated over 2 times. It was also noted that, IEC-6 cells secrete IL-7 cytokine into media when treated with *G.uralensis* polysaccharides. The secreted IL-7 stimulated proliferation of freshly isolated T lymphocytes within 6 h. The effect of the polysaccharides were found to be molecular weight depended, with low molecular weight having a profound effect compared to high molecular weight and total crude extract.

**Conclusion:**

Our findings indicate that *G.uralensis* polysaccharides especially those of low molecular weight have a potential as anticancer agents. Of great importance, is the ability of the polysaccharides to up-regulate anticancer cytokine IL-7, which is important in proliferation and maturation of immune cells and it is associated with better prognosis in cancer. Therefore, immunomodulation is a possible mode of action of the polysaccharides in cancer therapy.

**Electronic supplementary material:**

The online version of this article (doi:10.1186/s12906-016-1171-4) contains supplementary material, which is available to authorized users.

## Background

Cancer is a leading cause of death worldwide. According to Global cancer burden, there are estimated 12.7 million new cases and 7.6 million death, translating to 70 % increase in new cases and death [1]. It is projected that there will be an increase from 12.7 to 21.4 new cancer cases in the year 2030 [2]. In the United states, there were estimated1,665,540 new cancer cases and 585,720 cancer death in 2014 [3]. Cancer causes are complex but there are risk factors which contribute to its pathophysiology [4]. Due to this, cancer can be regarded to as a preventable disease since approximately 90–95 % of the cases are attributed to environmental factors and the remaining fraction is due to genetics. [4].

The prevention and management of cancer through surgery, radiotherapy, chemotherapy, hormonotherapy and complementary therapies have yielded varying results. Notably, conventional therapies lead to secondary complications especially metastasis, reduced immune competence, recurrence of tumors and decrease of quality of life (QOL) of patients [5–8]. Due to the varying effects of conventional treatments, lack of awareness and poverty, a great percentage of the world’s population uses alternative and complementary medicine for management and treatment of cancer [9, 10]. Among the complementary and alternative medicine, Traditional Chinese medicine (TCM), a holistic approach that focuses on improving body’s immune system to fight diseases, has been used to treat various diseases over thousands of years in East Asian countries. For instance, a number of different kinds of cancer have been treated with TCM drugs either alone or in combination with conventional therapies [11, 12]. *Glycyrrhiza uralensis* Fisch. is one of the oldest prescribed herb in Chinese traditional medicine for the treatment of various disease syndromes including cancer, by arresting tumorigenesis and metastasis [13–18]. *Glycyrrhiza uralensis* belongs to the Family Fabaceae (Leguminosae), genus *Glycyrrhiza* and species *Glycyrrhiza glabra, Glycyrrhiza lepidota, and Glycyrrhiza uralensis* found in Europe, Asia, Russia and Turkey where its roots are commonly used. It has phenolic compounds, triterpenes, saponins, flavonoids, polysaccharides, pectines and amino acids among others [19, 20].

Studies on *Glycyrrhizae* have shown potential of its compounds as anticancer and immune booster in vitro and in vivo. For instance, isoliquiritigenin reduces the multiplication of colon and lung cancer [21], glycyrrhizin and glabridin have anti-proliferative and apoptosis inducing properties against MCF-7 and HEP-2 cells [22, 23], chalconeisoliquiritigenin show antitumorigenic potential on prostate and breast cancer [24, 25], 18β-glycyrrhetinic acid up-regulates T cell proliferation and enhances the immune status by increasing blood leukocyte count and weight of the spleen when administered to mice [26–28].

The anti-cancer mechanism of Licorice compounds is not well known but it can be associated with augmenting T cell proliferation and modulating the immune system through stimulation and secretion of cytokine IL-7 which is associated with maturation, proliferation and maintenance of homeostasis of lymphocyte lineage cells. Research indicates that cytokines have a broad mechanism of action in cancer treatment. Due to this knowledge, a number of cytokines based therapies for cancer treatment have been developed. Among other cytokines, IL-7 has entered clinical trials for having a potential for adoptive immunotherapy in cancer therapy [29–31]. Interleukin 7 induces proliferation and long-term survival of freshly isolated T cells, Naïve and memory T cells, making it an essential cytokine in lymphopoeisis and homeostasis of T lymphocytes [32–34]

This study evaluated the antitumor and immunomodulatory potential of *G.uralensis* polysaccharides. The aim is to determine the effect of the polysaccharides on IL-7 gene expression, effect of IL-7 on proliferation of T lymphocytes and inhibitory potential of the polysaccharides on cancer cells.

## Methods

All chemical reagents were purchased from Sigma (Sigma-Aldrich Inc., St. Louis, MO, USA unless otherwise stated). Mouse Intestinal Epithelial cell line (IEC-6) and colon carcinoma cell line (CT-26) were provided by Institute of animal research in Beijing, China; Dulbecco’s Modified Eagle’s Medium (DMEM), Fetal bovine serum (FBS), Streptomycin/Penicillin and Insulin were purchased from Gibco, Beijing, China; synthetic oligonucleotides primers were purchased from Sangon Biotech (Shanghai) Co., Ltd). *Glycyrrhiza uralensis* polysaccharide extracts were provided by the Tianjin University ofTraditional Chinese Medicine(TUTCM) herbal pharmaceutical company, Tianjin, China. The polysaccharides were fraction A of over 100 kDa was 81.4 % (High molecular weight), fraction B, of 75 kDa was 45.4 and 54.6 % polysaccharide fractions was under 10 kDa (Low molecular weight) and fraction C, 34.5 % polysaccharide fractions was 290 kDa and 14 kDa was 30.3 %, total crude extract. Cell counting kit - CCK-8 (Dojindo Molecular Technologies, Beijing, China); Trizol (Invitrogen); QuantScript RT Kit (TIANGEN, Beijing, China); SuperRealPreMix SYBR Green (TIANGEN BIOTECH (BEIJING) CO.,LTD); Microtiterplate reader, VarioskanFlash.

### Cell lines and Cell culture

Mouse intestinal epithelial cell line (IEC-6) were routinely maintained in Dulbecco’s Modified Eagle’s Medium (DMEM) supplemented with 1 % streptomycin/penicillin (P/S), 1 % insulin and 10 % Fetal Bovine Serum (FBS) at 37 °C and 5 % CO_2_. Colon carcinoma cell line (CT-26) were routinely maintained in RPMI-1640 supplemented with 1 % streptomycin/penicillin (P/S), L- glutamine and 10 % Fetal Bovine Serum (FBS) at 37 °C and 5 % CO_2._ All cell lines were maintained in a humidified atmosphere. Standard cell culture protocols for cell culture were followed at the TUTCM laboratories.

### Antitumor and proliferative effect of *G. uralensis* polysaccharides

Proliferation and cytotoxicity of cells was determined using a CCK-8 assay (Dojindo Molecular Technologies, Beijing, China). A total density of approximately 5 × 10^3^ cells/well of IEC-6 and CT-26 were seeded in 96 well plates for 24 h. They were treated with different concentrations of Licorice polysaccharides at 10 μL per well and incubated at 37 °C, 5 % CO_2_ for 3 days. Subsequently, 10 μL of CCK-8 was added to each well, incubated in a high humidity environment at 37 °C and 5 % CO_2_ for 4 h. The absorbance was measured at a wavelength of 450 nm using a microtiter plate reader (VarioskanFlash using Skanlt software 2.4.3KE).

### IL-7 gene expression analysis by Real time polymerase chain reaction

To determine the immunomodulatory potential of *G. uralensis* polysaccharides through expression and secretion of IL-7, expression of IL–7 cytokine was evaluated by Real time PCR assay as previously described [35]. Briefly, IEC-6 cells were cultured in six well plates (2 × 10^5^) maintained in DMEM (Gibco) supplemented with various extracts at concentrations of 100 μg/ml at 3 and 72 h. After the treatment period, RNA was isolated using RNeasy total RNA extraction Kit (Qiagen, Beijing, China). cDNA was synthesised using QuantScript RT Kit (TIANGEN, Beijing, China) according to manufacturer’s instructions. Real time PCR assay was carried out using IQ5 Multicolor thermocycler (BIO-RAD Laboratories, Beijing) using SuperReal Premix SYBR Green I (TIANGEN BIOTECH (BEIJING) CO., Ltd) following manufacturer’s TIANGEN instruction kit. The synthetic oligonucleotides primers were; IL 7: (F) 5ʹ GAGTTTCAGACGGCACACAA 3ʹ and (R) 5ʹ GAAACTTCTGGGAGGGTTCC 3ʹ (product size: 229 bp) and GAPDH: (F) 5ʹ CCACAGTCCATGCCATCAC 3ʹ and (R) 5ʹ TCCACCACCCTGTTGCTGTA 3ʹ (product size: 452 bp). The PCR conditions and amplification efficiency for the genes were optimized at 95–98 %. Two step protocol was used, 95 °C for 15 min; 95 °C for 10 s and 60 °C for 32 s followed by Dissociation curve. Expression of the gene was analysed by the IQ5 Mutlicolor Real time PCR Software as the relative expression of IL-7 of GAPDH.

### Isolation of Lymphocytes and Lymphocyte activation assay

To determine secretion of IL-7 by IEC-6 cells treated with Licorice polysaccharides and proliferation of T cells stimulated by IL-7, Trypan exclusion assay and lymphocyte activation assay was done. Briefly, Rectal-orbital puncture method was used to draw blood from anaesthetised rat into anticoagulant treated tubes. A modification of lymphocyte isolation assay by density gradient centrifugation Ficoll-paque was used to isolate lymphocytes [36, 37]. The lymphocytes were cultured in supernatants of IEC-6 cells treated with *G.uralensis* polysaccharides, seeded in 24 well plates at a density of 1 × 10^4^ cells/ml and ratio of 1:1. As an internal control, IL-7 mouse antibodies were added to culture wells to block stimulation by IL-7 cytokine. After 6 h of incubation, the cell number and viability was determined by Trypan blue exclusion assay.

### Statistical analysis

Analysis of the results was done using SPSS statistical software version 16 and curve-fitting was done using GraphPadprism version 5. ANOVA was used to compare the means of different groups. Mean separation was carried out to determine the level of significance. The data are expressed as mean ± S.E.M. *P*-value of ≤ 0.05 was considered statistically significant.

## Results

### Proliferation of IEC-6 cells

**Fig. 1:**
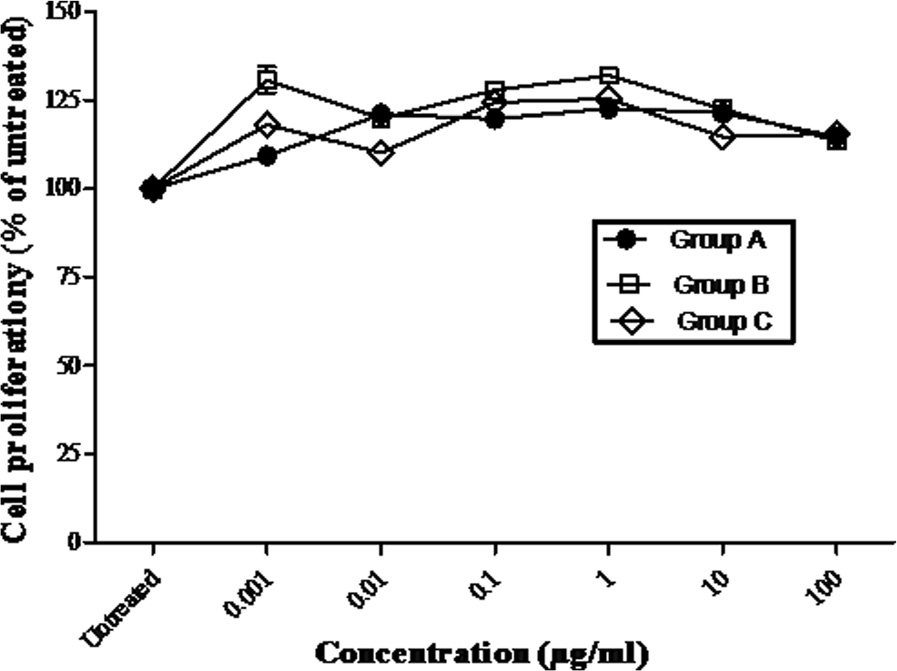
Effect of polysaccharides on IEC-6 viability and proliferation. IEC-6 cells (5 × 10^4^cells/ml) stimulated by polysaccharides (Fraction **a**, **b** & **c**) with concentration range of 0.001 μg/ml to 100 μg/ml for 72 h at 37°C, 5 % CO_2-_ Cell viability measured by CCK-8 assay. Data expressed as mean ± S.E.M of three tests done in triplicate

The proliferation and viability of IEC-6 cell line was measured by (CCK-8) assay. Proliferation of cells was determined as a percentage of viable treated cells in comparison to viable untreated controls. It was determined that *G.uralensis* polysaccharide extracts stimulated proliferation of IEC-6 cells in vitro. The optimum growth for IEC-6 cells was found to be 1 μg/ml, however, the ED_50_ was determined to be approximately 100 μg/ml. Interestingly, *Glycyrrhiza uralensis* polysaccharides stimulated proliferation of epithelial cells even at higher concentration of 100 μg/ml. Notably, there was no significant difference between proliferation activity of the three polysaccharide fractions, but, low molecular weight polysaccharides stimulated proliferation of epithelial cells more than the high molecular weight polysaccharides (Fig. 1). Furthermore, licorice polysaccharides showed little or no cytotoxic effects on the epithelial cells unless at obviously very high concentration.

### *G.uralensis* polysaccharides inhibits proliferation of colon carcinoma cell line (CT-26)

**Fig. 2:**
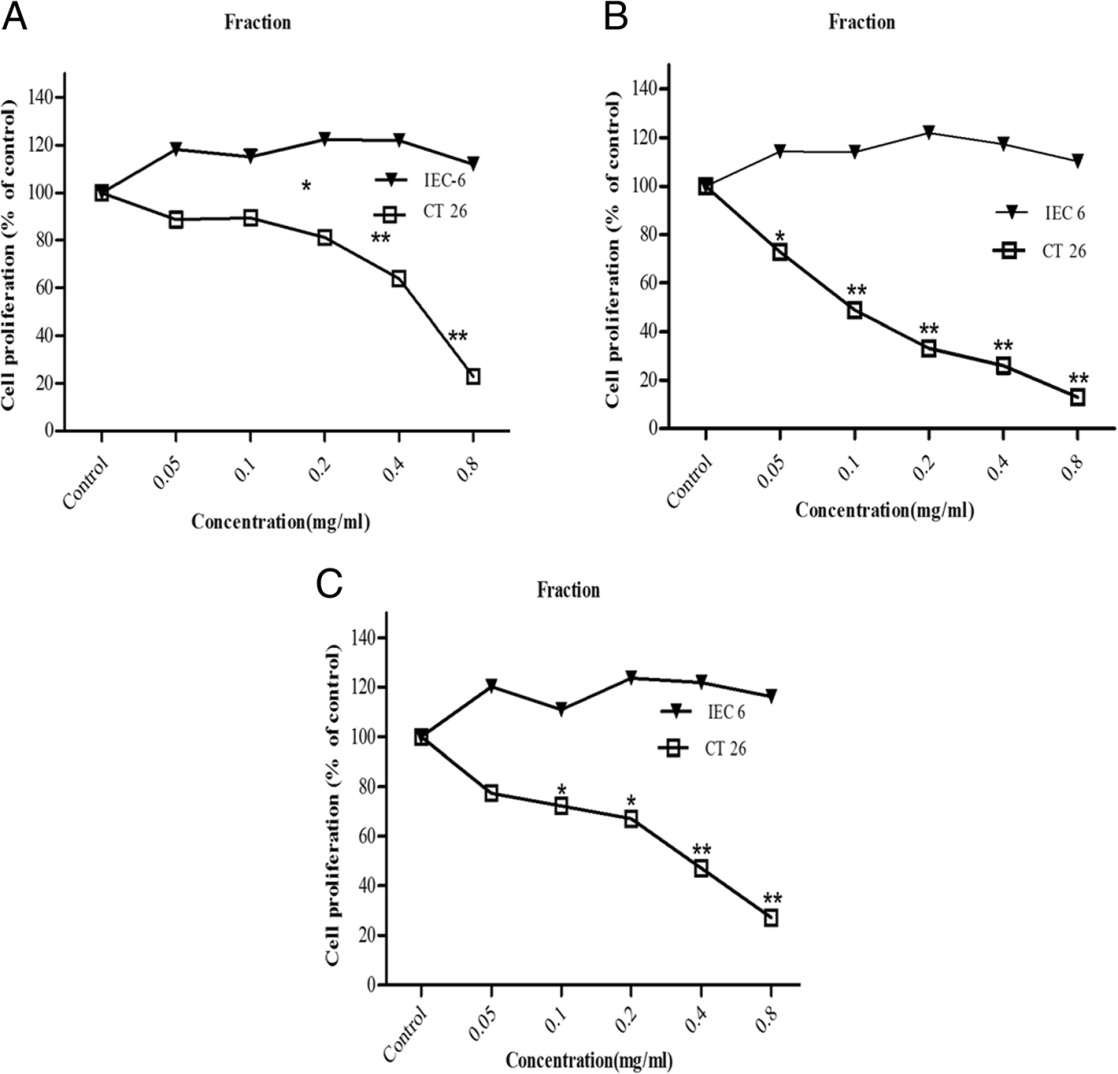
In vitro direct cytotoxic effect of Gancao polysaccharide extracts. Decrease in cell number was observed in CT 26 cells (5 × 10^4^ cells /ml) upon treatment with various concentration of polysaccharides (Fraction **a**, **b** and **c**) from 0.05 mg/ml to 0.8 mg/ml as measured by CCK-8 assay. Data expressed as mean ± SEM of three experiments. *P < 0.05, **P < 0.01 versus control

We investigated whether *G.uralensis* polysaccharides has the same effect of promoting proliferation of epithelial cells on cancer cell lines. Colon cancer cell line (CT-26) was treated with different concentrations of *G.uralensis* polysaccharides. The polysaccharides showed significant antitumor effects after 72 h. At a concentration of 0.05 mg/ml, growth inhibition was evident (Fig. 2) with nearly 50 % growth inhibition of cancer cells compared to epithelial cells, IEC-6, (Fig. 2). Comparing the three extracts, Fraction B, which is polysaccharide of lower molecular weight showed a higher inhibitory activity than the other fractions, A and C with more than 50 % of the growth inhibition of CT-26 cells at 0.1 mg/ml (Fig. 2). The results indicate that, while the licorice polysaccharides stimulate proliferation of normal cells (IEC-6), they inhibit growth of cancer cells even at low concentration.

### *G.uralensis* polysaccharides up-regulates expression of Interleukin 7

**Fig. 3:**
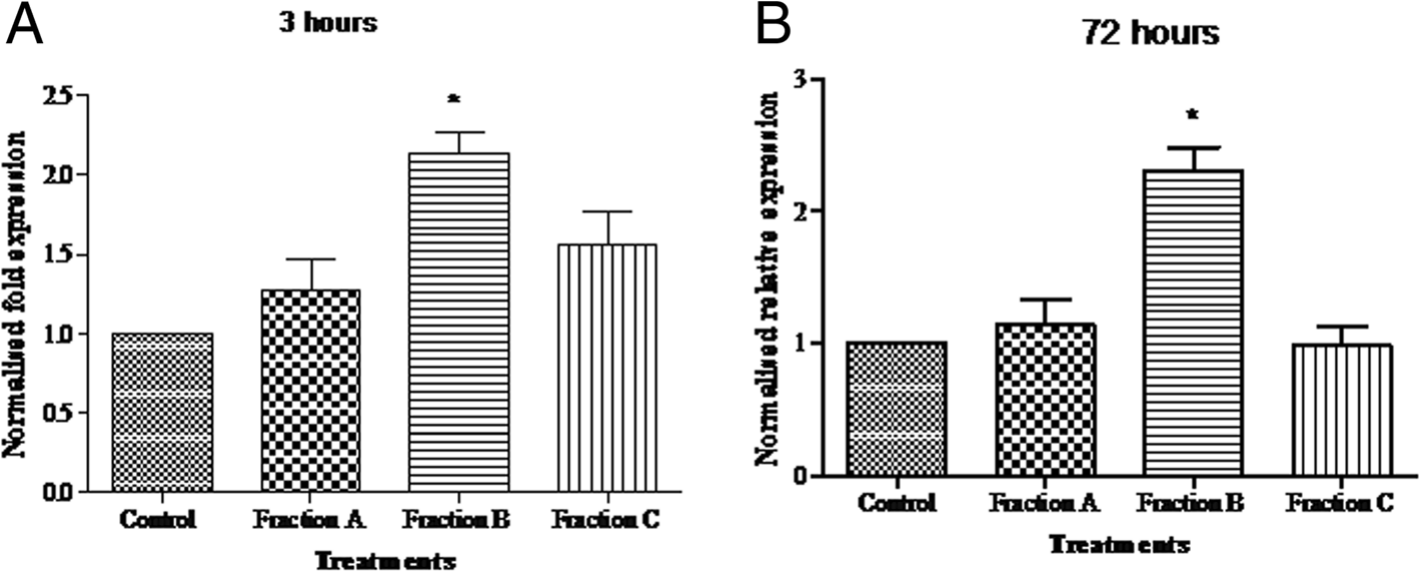
Expression of IL 7mRNA at (**a**) 3 h and (**b**) 72 h. 100 μg/ml of each polysaccharide fraction was added to IEC-6 cells and incubated for 3 and 72 h respectively. The normalized relative expression of IL-7 mRNA was analyzed by IQ5 Real-time PCR software. Data presented as mean ± S.E.M. *P < 0.05 versus untreated (control) of three experiments

To investigate the immunomodulatory effect of *G.uralensis* polysaccharides through upregulation of IL-7 gene, the expression level of IL-7 in licorice polysaccharide treated IEC-6 cells was determined. The IEC-6 cells treated with *G.uralensis* polysaccharides showed up-regulation of the IL-7 gene after 3 h and the upregulation remained elevated upto 72 h of treated. The up regulation of these cytokine was depended on the molecular weight of the extracts, going up by over two times with fraction B (Fig. 3). The up-regulation of the cytokine was not time dependent, at 3 and 72 h, the up-regulation was not significantly different (Fig. 3a and b).

### Interleukin 7 enhances T lymphocyte proliferation

**Fig. 4:**
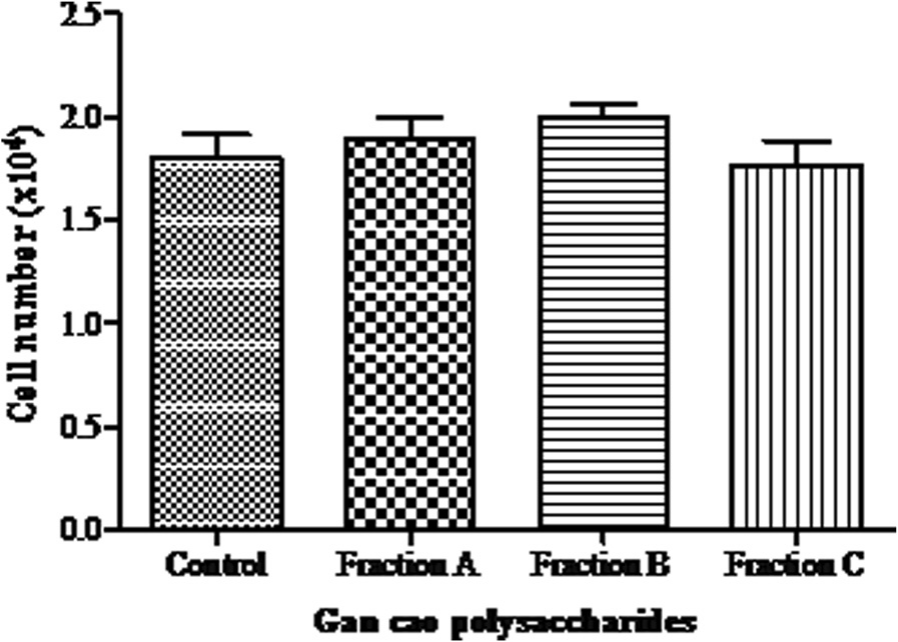
Activation and proliferation of T lymphocytes by secreted IL-7 Freshly isolated T lymphocytes maintained in supernatants of IEC-6 polysaccharide treated cells. Data represented as mean ± S.E. of two experiments

We sought to establish whether the activation and proliferation of T lymphocytes was due to secreted interleukin 7. After a 6 h incubation of the T lymphocytes with various supernatants derived from IEC-6 cells treated with *G.uralensis* polysaccharides, the T lymphocytes number was determined as a measure of activation and proliferation. From the results, all supernatants of IEC-6 cells treated with *G.uralensis* polysaccharide showed a slight increase in number of T lymphocytes (Fig. 4). Low molecular weight polysaccharide Fraction B, showed a higher proliferation activity among the three fractions, though there was no significant difference between these fractions.

## Discussion

This study focused on determining the anticancer potential of polysaccharides from Chinese licorice which is a major guiding drug in many Chinese herbal prescriptions. Immunomodulation has been recognised as an important molecular mechanism targeting control of cancers especially by use of cytokines.

*Glycyrrhiza uralensis* has been used and fronted as an anticancer agent. Apart from its pharmacological application, Gancao, as known in China, has been used as a flavoring agent and sweetening agent in tobaccos, chewing gums, candies, and toothpaste among other uses [38, 39]. *G. uralensis* is used as a flavouring agent to disguise the unpleasant taste and smell of other drugs. Apart from the medicinal properties, Gan cao is used as food, a reason that can be attributed to the non toxicity of its polysaccharides on gut mucosal cells. This was confirmed from our study where the Gancao polysaccharides showed no cytotoxic effects on normal gut cells, IEC-6 cells (Fig. 1). It was observed that the polysaccharides fraction from Gan cao stimulated proliferation of Intestinal epithelial cells. These polysaccharides are therefore safe for consumption both as dietary suppliments or food and herbal prescriptions.

*G. uralensis* is used in the treatment of various diseases [40]. In Japan and China, apart from its pharmaceutical application, it is used as a sweetening and flavouring agent in pharmaceutical industries. It is used to treat various ailments and diseases including cancer. The mechanism of action in cancer treatment are varied including apoptotic cell death through upregulation of p53 and p21, cell cycle arrest, suppression of nitric oxide production among others [41–44]. The antitumor activity of Gan cao was evident when tumor cells (CT-26 cells) were exposed to these polysaccharides. The cell growth was inhibited even at low concentration of 0.05 mg/ml. The inhibition was dependent on the molecular weight of the polysaccharides with low molecular weight polysaccharides showing a higher growth inhibition compared to the other polysaccharides (Fig. 2). It is therefore paramount to link Gan cao polysaccharides to direct activity on tumor cells. This antitumor activity is true for polysaccharides especially low molecular weight polysaccharides which have been proven to have immunomodulation and antitumor activity [45]. Though it has been shown that polysaccharides do not have direct effect on cancer cells (Wasser 2002), our study showed that Licorice polysaccharide directly inhibited growth of CT-26 tumor cells but not IEC 6 cells in vitro at a concentration of 0.05 mg/ml. Fraction B of low molecular weight inhibited proliferation of CT-26 cells in a dose-dependent manner and at less than 0.1 mg/ml concentration, its cytotoxicity effect was half in comparison to other fractions. The results concurred with earlier findings confirming the antitumor potential of Gan cao polysaccharides [46–48].

Gan cao has previously been associated with anticancer, antioxidant and anti-inflammatory effects. Among its mode of action, immunomodulation has been exhibited by Gancao [42, 49, 50]. The effect of Gan cao polysaccharide on IL-7 has not been explored. IL-7 is an important cytokine in cancer immunotherapy and it has potential for adoptive immunotherapy [51]. Interleukin 7 homeostasis determines host survival and is an indicator of host immunocompetence. Increased levels of IL-7 leads to T cell activation and lack of IL-7 leads to severe immunodeficiency [52, 53]. A part from bone marrow and other sites of production, intestinal epithelial cells have recently been identified as a major site of secretion [54–56]. From our experiments, epithelial cells cultured with licorice polysaccharides up-regulated the expression of IL-7 cytokine. The expression was elevated at 3 h and remained elevated up to 72 h by two times compared to the untreated controls (Fig. 3). Augmented expression of IL-7 translates to increased circulation of IL-7 protein. IL-7 produced in the gut initiates local immune response and promote overall immune activity to pathogens through stimulation and maturation of T lymphocytes [57]. Cytokines like IL-7, when secreted, stimulate immune effector cells and enhance tumor cell recognition by cytotoxic effector cells [31]. The elevated expression of IL-7 cytokine therefore is an indicator of the immunomodulatory potential of Gancao polysaccharides. Earlier research has shown, in vitro and in vivo, IL-7 is responsible for development, maturation, proliferation and homeostasis of T lymphocytes [58, 59]. These findings are in agreement with our results, in which freshly isolated T lymphocytes cultured in supernatants containing secreted IL-7, though to a slight extend, enhanced proliferation of lymphocytes. We can therefore confirm that, IL-7 secreted by IEC 6 cells treated with Gan cao polysaccharides can enhance proliferation of T lymphocytes (Fig. 4). The proliferation of T lymphocytes leads to CD4^+^ and CD8^+^ infiltration of tumors which is associated with better prognosis [60]. Furthermore, histology of tumors from IL-7–treated mice show heavy infiltration with both CD4^+^ and CD8^+^ T cells [61]. Protection against tumor progression and metastasis is attributed to cellular immunity due to CD4^+^ nad CD8^+^ cells [62]. Population and function of tumor specific CTL are enhanced by presence of tumor specific CD4^+^ T cell responses, their lack leads to tumor progression and abrogates the survival of tumor bearing hosts. It involves CD8^+^ as key effector cells of which CD4^+^ plays an important role in their production, expansion maintenance and activation. The end results of combined CD4^+^ and CD8^+^ is better than one, though CD4^+^ is essential for effective antitumor CTL responses by enhancing their number and function. In addition to enhancing responses of CD8^+^ T cell responses, CD4^+^ mediate tumor rejection through cytotoxic effect on tumor cells, up regulation of MHC molecules expression, inhibition of angiogenesis and induction of tumor dormancy [63, 64]. It is therefore paramount that the upregulation of IL-7 leads to augmentation of T lymphocytes which are important in cancer immunotherapy.

## Conclusion

A major finding of our research is that licorice polysaccharides have an antitumor and immunomodulatory activity. They stimulate proliferation of IEC-6 cells which are major sites of IL-7 production. These polysaccharides have a direct antitumor potential as they inhibit proliferation of tumor cells, CT-26, even at low concentration. Immunomodulatory potential of these polysaccharides is exhibited through up-regulation of the relative expression of IL-7 gene, the immune cytokine, IL-7. This cytokine has been fronted for enhancement, maturation, proliferation and long survival of immune effector T cells. Finally, Gan cao polysaccharides can enhance secretion of IL-7 cytokine by IEC-6, which subsequently indicates that this cytokine has the potential of promoting proliferation of T lymphocytes. From these finding, we can therefore propose that Gan cao polysaccharides have direct anticancer potential on tumor cells and also have immunomodulatory potential through upregulation of IL-7 cytokine, which research has shown to play a major role in cancer immunotherapy.

## Abbrevations

ANOVA, analysis of variance; CCK, cell counting kit; CD, cluster of differentiation; cDNA, complementary deoxyribonucleic acid; CO_2_, carbon dioxide; DMEM, Dulbecco’s Modified Eagle’s Medium; ED, effective dose; FBS, fetal bovine serum; GAPDH, glyceraldehyde-3-phosphate dehydrogenase; IEC, intestinal epithelial cells; IL, interleukin; kDa, kilodalton; P/S, penicillin/streptomycin; PCR, polymerase chain reaction; RNA, ribonucleic acid; RPMI, Roswell Park Memorial Institute; SEM, standard error mean; TCM, traditional Chinese medicine; TUTCM, Tianjin University of Traditional Chinese Medicine

## Additional file

Additional file 1: Availability of data and materials. (XLS 40 kb)
